# Exploring potential impacts of pregnancy-related maternal immune activation and extracellular vesicles on immune alterations observed in autism spectrum disorder

**DOI:** 10.1016/j.heliyon.2023.e15593

**Published:** 2023-04-29

**Authors:** Valéria de Lima Kaminski, Rafael Tomoya Michita, Joel Henrique Ellwanger, Tiago Degani Veit, Jaqueline Bohrer Schuch, Rudimar dos Santos Riesgo, Tatiana Roman, José Artur Bogo Chies

**Affiliations:** a Laboratório de Imunobiologia e Imunogenética, Departamento de Genética, Universidade Federal do Rio Grande do Sul – UFRGS, Porto Alegre, Rio Grande do Sul, Brazil; b Programa de Pós-Graduação em Genética e Biologia Molecular, Departamento de Genética, Universidade Federal do Rio Grande do Sul – UFRGS, Porto Alegre, Rio Grande do Sul, Brazil; c Programa de Pós-Graduação em Biotecnologia, Laboratório de Imunologia Aplicada, Instituto de Ciência e Tecnologia - ICT, Universidade Federal de São Paulo - UNIFESP, São José dos Campos, São Paulo, Brazil; d Laboratório de Genética Molecular Humana, Universidade Luterana do Brasil - ULBRA, Canoas, Rio Grande do Sul, Brazil; e Instituto de Ciências Básicas da Saúde, Departmento de Microbiologia, Imunologia e Parasitologia, Universidade Federal do Rio Grande do Sul - UFRGS, Porto Alegre, Rio Grande do Sul, Brazil; f Centro de Pesquisa em Álcool e Drogas, Hospital de Clínicas de Porto Alegre, Universidade Federal do Rio Grande do Sul - UFRGS, Porto Alegre, Rio Grande do Sul, Brazil; g Programa de Pós-Graduação em Psiquiatria e Ciências do Comportamento, Universidade Federal do Rio Grande do Sul – UFRGS, Porto Alegre, Rio Grande do Sul, Brazil; h Child Neurology Unit, Hospital de Clínicas de Porto Alegre, Universidade Federal do Rio Grande do Sul - UFRGS, Porto Alegre, Rio Grande do Sul, Brazil

**Keywords:** Autism spectrum disorder, Immune system, Infection, Inflammatory response, Gestation, Cytokines, Maternal immune activation, Preeclampsia, Microbiota, Exosomes

## Abstract

Autism Spectrum Disorder (ASD) is a set of neurodevelopmental disorders usually observed in early life, with impacts on behavioral and social skills. Incidence of ASD has been dramatically increasing worldwide, possibly due to increase in awareness/diagnosis as well as to genetic and environmental triggers. Currently, it is estimated that ∼1% of the world population presents ASD symptoms. In addition to its genetic background, environmental and immune-related factors also influence the ASD etiology. In this context, maternal immune activation (MIA) has recently been suggested as a component potentially involved in ASD development. In addition, extracellular vesicles (EVs) are abundant at the maternal-fetal interface and are actively involved in the immunoregulation required for a healthy pregnancy. Considering that alterations in concentration and content of EVs have also been associated with ASD, this article raises a debate about the potential roles of EVs in the processes surrounding MIA. This represents the major differential of the present review compared to other ASD studies. To support the suggested correlations and hypotheses, findings regarding the roles of EVs during pregnancy and potential influences on ASD are discussed, along with a review and update concerning the participation of infections, cytokine unbalances, overweight and obesity, maternal anti-fetal brain antibodies, maternal fever, gestational diabetes, preeclampsia, labor type and microbiota unbalances in MIA and ASD.

## Highlights

• Environmental factors influence the etiology of ASD.
• Maternal immune activation (MIA) during pregnancy can contribute to ASD development.
• Extracellular vesicles could contribute to immune impairments observed in ASD patients.

## 1 Introduction

Maternal immune activation (MIA) during pregnancy encompasses a set of immune unbalances at the maternal-fetal interface suggested to be risk factors for Autism Spectrum Disorder (ASD) development in the newborn [1]. Extracellular vesicles (EVs) are important players in immuoregulation, affecting multiple physiological processes [2]. In this sense, EVs released by the placenta are abundant at the maternal-fetal interface and actively participate in the immunoregulation required for a healthy pregnancy [3]. Alterations in the content and levels of EVs in the maternal circulation are associated with gestational disorders [4], and some evidence has also associated altered levels of EVs with ASD [5]. Considering these findings, this narrative review raises potential impacts of EV-related immune alterations on (I) ASD incidence when addressed during pregnancy, and (II) ASD symptomatology when such alterations on EV levels and cargos are detected in patients. Physiological and immune processes related to these topics are also reviewed and discussed.

ASD is a set of heterogeneous neurodevelopmental disorders that impact behavioral and social skills since early life. ASD is approximately four times more common among boys than among girls, and 1% of the overall world population is estimated as presenting symptoms that could predispose to ASD diagnosis. Once considered a rare condition with a prevalence of 4–5/10,000, autism today has a prevalence ranging from 0.9 to 1.5% [6]. Since 1998 the Centers for Disease Control and Prevention (CDC) tracks ASD prevalence and the characteristics of children with ASD in the United States. In 2000, CDC established the Autism and Developmental Disabilities Monitoring (ADDM) network as an attempt to track ASD prevalence. According to ADDM, ASD prevalence in eight-year-old children in different communities of the United States showed a dramatic increase, changing from one diagnostic in 150 children during the 2000–2002 period to one in 68 during 2010–2012 [7].

ASD has a strong genetic component, showing a high concordance rate between monozygotic twins. However, the exact causes of ASD are still unknown. Along with genetics, environmental factors are probably the primary triggers of ASD, especially considering that the ASD incidence rate is not fully concordant in monozygotic twins [1,8,9]. In the meta-analysis performed by Ref. [10], the concordance rate in monozygotic twins was almost complete (98%), being lower for dizygotic twins (53–67%). According to these findings, a substantial meta-analytic heritability of 64–91% was estimated. On the other hand, the discordance rate in the ASD incidence in cases of monozygotic twins brings to light the role of environmental triggers for ASD development and manifestation.

**Fig. 1:**
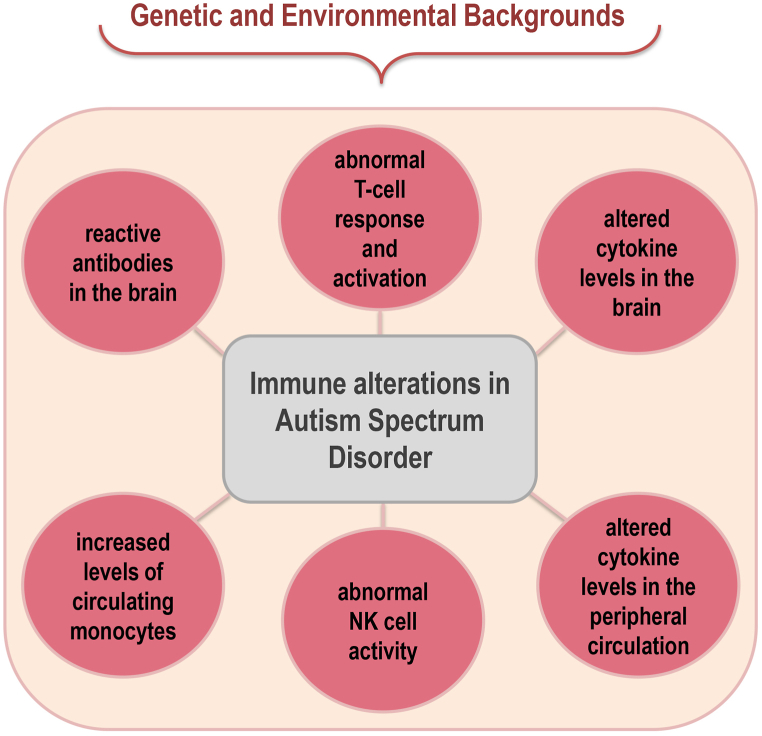
Most commom immune alterations observed in Autism Spectrum Disorder (ASD).

It is known that the immune system influences both the development and severity of ASD due to interactions with the central nervous system (CNS) [11]. Fig. 1 briefly presents the immune alterations observed in ASD patients (more discussion on these aspects can be found in section 3 and the following sections). In this sense, MIA is a critical risk factor for ASD development due to the exposure of the fetus to inflammatory mediators during pregnancy [1], as will be discussed in detail in section 5. Although inflammation plays a crucial role during embryo implantation allowing the remodeling of blood vessels and tissues [12], pro-inflammatory responses are not systemically predominant during a healthy gestational period; instead, there is a constant immunomodulation, mainly at the maternal-fetal interface, which allows fetal development and provides protection against pathogens [4].

The role of infections during pregnancy and the development of ASD has been a main target of investigation, as summarized in Table 1 [[13], [14], [15], [16], [17], [18], [19], [20], [21], [22]]. A possible link between maternal infection during pregnancy and autism was initially raised in a study addressing rubella syndrome in 1950, in which it was observed that 5% of the infected pregnant woman had children with ASD [13,14,23]. Subsequently, additional studies suggested that other pathogens, including influenza and measles viruses, could also be contributing factors to ASD development. After the first associations between autism and viral infections during pregnancy, it was believed that ASD was directly associated with infections *per se*. Nevertheless, the incidence of ASD in children born from women with complicated pregnancies, not related to infections, suggests an association between ASD and maternal inflammation in general (i.e. inflammation induced by different triggers) as a more likely explanation for these initial observations [24].

**Table 1:** Infections during pregnancy or in newborns investigated in the context of ASD.

Pathogen or infection investigated	Country	Sample characterization	Data on pathogen exposure	Main findings	Reference
Rubella virus	USA	Following the 1964 rubella outbreak, 243 children with congenital rubella syndrome were examided	All samples were part of a follow-up study.	Congenital rubella was associated to autism.	[13,14]
CMV	USA	Mothers of 82 children with ASD symptoms were tested for CMV IgG and HSV2 IgG in serum.	Samples were tested for CMV IgG and HSV2 IgG in serum.	Maternal CMV infections may influence ASD symptoms.	[15]
Influenza virus	USA	538 children with ASD, 163 with developmental delays, and 421 typically developing controls.	Maternals interviews.	No association was found.	[16]
Influenza virus infection; Vaginal yeast infection; Genital herpes; Labial herpes	Denmark	96,736 children from a population-based cohort where 976 children (1%) were diagnosed with ASD.	Self-reported of the mothers through telephone interviews during pregnancy and early postpartum.	An increased risk of autism in the child after self-reported infection with influenza virus during pregnancy was observed.	[17]
Influenza, Chickenpox, mumps and rubella infections	USA	163 cases of autism and 355 respective non-diagnosed siblings.	Clinical records and parents' interviews were used for data collection.	Exposure to rubella, mumps and chickenpox during gestation was associated with cases of autism.	[18]
Zika virus	Brazil	216 infants followed since the 2015–2016 ZIKV epidemic in Rio de Janeiro.	PCR-confirmed maternal ZIKV infection in pregnancy.	Three children were diagnosed with ASD.	[19]
Different neurotropic/polyomaviruses: CMV, EBV, HSV1, HSV2, HHV6, BKV, JCV, and SV40	USA	Postmortem brain tissue from 15 autistic patients and 13 controls.	Nested PCR followed by DNA sequence analysis.	BKV, JCV, and SV40, either singly or in combination, were significantly more frequent in autistic-derived brain tissues.	[20]
Two categories: organism-specific infections (viral, bacterial, mycosal, parasitic, unknown); organ-specific infections (cardiovascular, ear, eye, gastrointestinal, genitourinary, lower respiratory, upper respiratory, skin, other, unknown).	USA	The study population was drawn from the Childhood Autism Perinatal Study among the membership of Kaiser Permanente of Northern California.	Data were extracted from Kaiser Perm Northern California clinical databases.	No overall association between diagnoses of any maternal infection during pregnancy and ASD was observed.	[21]
Cytomegalovirus	Japan	Two case reports.	Giant cell analysis in urine and serology for case 1. Serology and PCR test in case 2.	Case 1: Giant cell with intranuclear inclusions characteristic of CMV was found in the urine 2 days after birth. Serum CMV-specific IgM antibodies were positive.	[22]
				Case 2: Serum CMVspecific IgM antibodies were positive, and PCR revealed the presence of CMV-DNA in the urine.	

ASD: Autism Spectrum Disorder; CMV: Cytomegalovirus; EBV: Epstein-Barr virus; BKV: BK virus; HHV6: Human herpesvirus 6; HSV1: Herpes simplex virus 1; HSV2: Herpes.

**Fig. 2:**
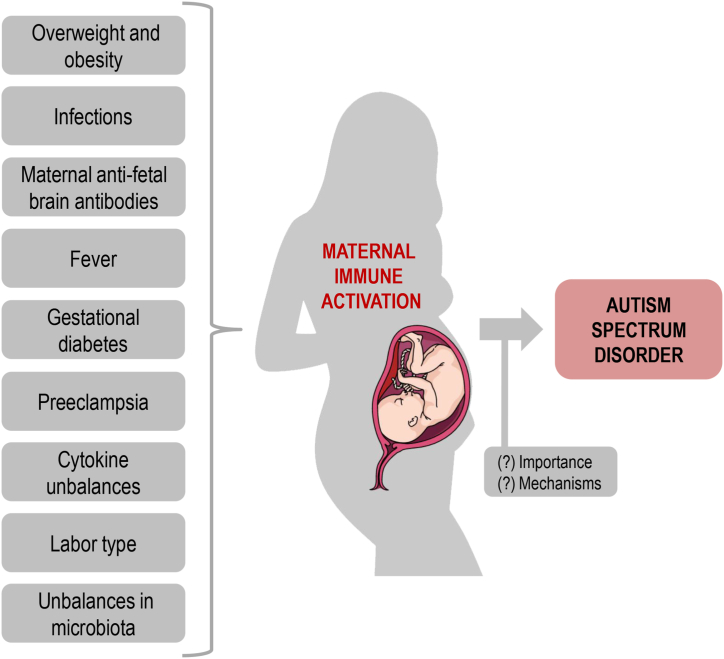
Factors involved in maternal immune activation (MIA) and potentially involved in the development of autism spectrum disorder (ASD). Overweight and obesity, infections, maternal anti-fetal brain antibodies, fever episodes, gestational diabetes mellitus, preeclampsia, imbalances in cytokine systems, labor type, and unbalances in maternal microbiota are factors associated with MIA. The development of ASD in the children can be, at least in part, the result of MIA during the gestational period. The degree (importance) of MIA and the mechanisms by which MIA affects the etiology of ASD must be established in greater detail. This figure was created using a *Mind the Graph* illustration (available at www.mindthegraph.com).

Gestational disorders characterized by inflammatory unbalances, such as preeclampsia, lead to severe consequences for both the mother and the fetus [25,26]. As depicted in Fig. 2, these and other gestational disturbances might be critical environmental triggers of inflammatory responses that ultimately affect the fetus developing brain, potentially leading to ASD development [1].

Throughout this manuscript, we mention studies involving animal models of ASD. We encourage the readers to search for details regarding the ASD models used in these studies, since some limitations in extrapolating data from models to the clinics are intrinsically related to the models used. In short, many scientific efforts have been made in the last years to create new models to study ASD, both *in vitro* and *in vivo*. Such models have the potential to help to validate some of the previously associated risk factors to ASD development, as well as to test new potential therapies to alleviate ASD symptoms [27]. *In vitro*, Shi et al. [28] presented techniques that allow the transformation of differentiated cell lines into induced pluripotent stem cells (hiPSCs). This is an exciting possibility for future studies in neurodevelopmental disorders, since hiPSCs can be differentiated in different cell types of the nervous system. Besides, cellular models directly derived from patients have also been addressed with promising results for studying molecular mechanisms of ASD [29].

Regarding animal models, they are considered adequate when such models show strong similarities with human phenotypes; the same biological abnormality as the cause of the disease as well as similar responses to therapies that could benefit humans with the analogous disorder [27]. The most used animal models in neuroscience research are rodents, being *Mus musculus* the most frequent species. However, other model organisms such as *Rattus norvegicus* or *Danio rerio* are also emerging, thanks to the evolution of genome sequencing and other techniques [[30], [31], [32]]. Research with *Rhesus* monkeys has also been conducted [33], as will be cited forward.

In this article, we summarize the main triggers and enhancers of inflammatory responses during pregnancy that have been associated with or suggested as significant risk factors for ASD development. Specifically, a review and an update regarding the participation of infections, cytokine unbalances, overweight and obesity, maternal anti-fetal brain antibodies, maternal fever, gestational diabetes, preeclampsia, labor type, and microbiota unbalances in the development of MIA and ASD are performed. Besides, emerging trends in the field of EVs and their impacts on mental and psychiatric diseases, including ASD, are reviewed.

## 2 Methods

This is a narrative review. The documents included in this review were retrieved from online research platforms, including PubMed (https://pubmed.ncbi.nlm.nih.gov/), Google Scholar (https://scholar.google.com/), and SciELO (https://www.scielo.br/). Also, some articles were selected based on the articles' reference list and the authors’ reference archive. The results and discussion of each topic addressed in this review will be presented below.

## 3 Inflammatory alterations observed in ASD

Considering the polygenic [34] and epistatic [35] genetic counterparts in ASD etiology, it is proposed that environmental factors interact with the human genetic background, ultimately increasing the disease risk or the severity of the symptoms observed throughout the disease spectrum [36,37]. Impairment in inflammatory responses is a characteristic feature in ASD patients during life, indicating a strong immune component in the disease manifestation [[38], [39], [40]]. The immune component of ASD was suggested to mainly rely on the dis-regulation of inflammatory processes, both during pregnancy (presented in the next sections) as well as later in life, as a trigger to a part of the symptoms.

Several discoveries have associated ASD with alterations in the immune system [41]. As summarized in Fig. 1, such alterations include the presence of brain-reactive antibodies [42]; abnormal T cell responses, proliferation, and activation [43,44]; altered brain, cerebrospinal fluid (CSF) and peripheral blood cytokine levels [[45], [46], [47]]; increased levels of circulating monocytes [48]; and dysregulation in Natural Killer (NK) cells activity [49]. Regarding NK cells, adults with high-functioning autism have elevated levels of NK cell activation along with other alterations in NK cell functioning, including over-expression of the NKG2C receptor [50].

Ross et al. (2013) [51] examined the circulating cytokine levels in DiGeorge syndrome patients, a condition related to an increased risk for autism and schizophrenia. In such study, a correlation between social impairments and elevated levels of the pro-inflammatory cytokines interleukin (IL)-1β and IL-6, as well as the anti-inflammatory cytokine IL-10 was observed. Furthermore, elevated levels of Th1 cytokines IL-12p70 and interferon (INF)-γ were associated with social and repetitive behavior scores. Increased levels of pro-inflammatory cytokines and chemokines such as IFN-γ, IL-1β, IL-6, IL-12p40, tumor necrosis factor (TNF)-α, and chemokine C–C motif ligand (CCL)-2 were also observed in the brain tissue and CSF in ASD patients [45,52], which could affect functions of the immune system at CNS. Besides, elevated IL-16 expression was already associated with the development of immune dysfunction in children with autism, and IL-16-expressing CD4^+^ and CD8^+^ T lymphocyte numbers were higher in children with ASD compared to typically developing controls [53].

Cytokines and their receptors are highly implicated in inflammatory processes, CNS development, neuronal plasticity, and the regulation of a number of cellular pathways at the transcription level [54,55]. In children with ASD, an upregulation of peripheral CXC and CC chemokine receptor expression on CD4^+^ T cells was already associated with immune dysregulation [56]. Besides, dysregulation in IL-6 receptors was associated with upregulated IL-17A-related signaling in CD4^+^ T cells of ASD children [57]. However, it is also important to call attention to the role of immune-related transcription factors signaling pathways involved in ASD, once it was already demonstrated in a BTBR T + Itpr3tf/J mouse model of autism that adenosine A2A receptor modulates neuroimmune function through the transcription factor Th17/retinoid-related orphan receptor gamma t (RORγt) signaling [58].

Concerning immune alterations as contributors to disease progression, some peripheral inflammatory markers were addressed in the context of ASD-related comorbidities and clinical findings. Results showed elevated levels of IL-1β, IL-6, IL-17, IL-12p40, and IL-12p70 cytokines in individuals with ASD compared with age-matched typically developing controls [59]. In this same study, all ASD patients showed interictal epileptiform activity at electroencephalography record. However, only 37.5% of the patients suffered from epilepsy. Also, higher IL-6 levels were observed in patients without a history of epilepsy with interictal epileptiform activity in the frontal brain region, reinforcing that peripheral inflammatory molecules could be potential biomarkers to predict comorbidities in ASD [59]. In this sense, alarmins, a heterogeneous group of proteins with different functions, were also approached as possible prognostic markers in ASD. These molecules are released by different cells in the surrounding tissues following cell damage or inflammation. Studies have shown that alarmins like interleukin IL-33, high-mobility group box 1 (HMGB1), heat-shock proteins (HSP), and S100 protein could play a relevant role in the pathogenesis of ASD. Moreover, it was also suggested that other alarmins, which block pro-inflammatory mediators, could ameliorate ASD symptoms [60].

## 4 Maternal infections that reach the fetus

During the gestational period, there is an intimate contact (although not a mixture) between maternal and fetal blood. This contact is tightly regulate by immunomodulatory factors produced by the placenta, which avoid infections and maternal immune responses towards the fetus [4]. However, some pathogens commonly bypass placental defenses and reach the fetus, sometimes causing severe and undesirable effects. The most common pathogens, considering pregnancy-related infections, are collectively known as TORCH [*Toxoplasma* sp., “other,” Rubella virus, Cytomegalovirus (CMV), and Herpes simplex virus (HSV)]. Nevertheless, other pathogens are also capable to cross the placenta and affect fetal development, Zika virus (ZIKV) being an impressive example of a virus presenting such capacity [[61], [62], [63]].

In addition to the classic effects, especially congenital malformations, caused by the abovementioned pathogens, some studies have associated infections during pregnancy with ASD development in the first years of life [[14], [15], [16],^19^]. Stella Chess described the first association between ASD and infections during pregnancy, through a study involving children with congenital rubella after the rubella epidemic in the United States in 1964 [13]. Five years later, a longitudinal study conducted with the same cohort, reinforced this association [14]. As previously mentioned, rubella virus and CMV are part of the TORCH group of pathogens, and infections with these agents during pregnancy have been associated with ASD development [15]. Furthermore, ZIKV infection during pregnancy was strongly associated with the development of microcephaly - characterizing the congenital Zika syndrome, a condition also described as a risk factor for ASD [19]. Importantly, the absence of microcephaly does not abrogate the risk of neurological problems in children of mothers with gestational ZIKV infection [64].

As a matter of fact, pregnant women with one or more episodes of infection with different pathogens have a higher risk of having children with ASD. An illustrative case-control study with a large sample size including 407 cases and 2075 frequency-matched controls revealed that women with one or more episodes of infection during pregnancy presented a higher risk of having children who would be later diagnosed with ASD. Specifically, this association was true in cases of bacterial infection during the second trimester of pregnancy [21]. This finding corroborated a previous study by Ref. [17] who found that hospitalization of pregnant women due to viral infection in the first trimester, and due to bacterial infection during the second trimester, were both later associated with the diagnosis of ASD in the offspring.

As previously mentioned, Table 1 summarizes the different types of infections during pregnancy that have already been investigated as possible risk factors for ASD development. All findings discussed here cover studies focusing on infections that occur during pregnancy and their association with ASD. Other studies also investigated possible associations between particular infections in childhood or adulthood and ASD, such as HSV [65] and Varicella zoster virus [66]. There are few data on these aspects for conclusions regarding the potential impact of infections in childhood or adulthood and ASD development to be drawn. However, taking together, these studies suggest that MIA due to infections could contribute to ASD development.

## 5 Maternal immune activation (MIA)

In addition to the most evident scenario showing that some infectious agents have a direct impact on neurodevelopment processes, several pieces of evidence point to indirect effects of infections during pregnancy and ASD risk as well as on the neurological development in the offspring. MIA refers to maternal immune dysregulation in response to prenatal insults. Such dysregulation often involves maternal infections and the subsequent immunological activation, but MIA can also refers to an activated immune response in the mother in the absence of any evident direct infection [1]. In this alternative scenario, maternal infections during pregnancy, with pathogens unable to reach the fetus, would lead to the activation of maternal immune responses. These responses, through immune mediators (e.g. cytokines, antibodies) or physiological alterations (e.g. fever) would impact the fetus' neurodevelopment, imposing the risk for the development of ASD and other neurological diseases.

One question still to be addressed is whether MIA alone would be sufficient to trigger autistic behaviors in the offspring. In this regard, an experiment using a mouse model of MIA during mid-pregnancy, either through influenza infection or the administration of TLR3 agonist poly (I:C), led to abnormal behavioral responses of the offspring as adults, such as deficits in prepulse inhibition (PPI) in the acoustic startle response and deficiencies in social interaction [23]. A later study from the same research group analyzed the cerebellum of these animals and found a localized deficit of Purkinje cells, a common finding in individuals with schizophrenia and ASD [67]. Therefore, it is possible that inflammatory mediators released from immune cells during the infection episodes could negatively influence brain development, resulting in ASD. Furthermore, a study using an MIA swine model suggested that fetal microglia is significantly altered by maternal infection with porcine reproductive and respiratory syndrome virus, which suggests a potential mechanism through which MIA could directly impact prenatal brain development and function [68], notwithstanding the limitations associated to the data obtained from animal studies. In fact, findings from studies with animal models should be interpreted with caution since differences in placentation and duration of gestation, as compared to humans, are not taken into account in such studies. That said, and being careful with extrapolations, studies with animal models still provide interesting insights on the possible impacts of MIA on ASD development in humans.

Thus, in addition to the hypothesis of placental barrier affected by infection, activation of the mother immune system as a consequence of the presence of infections potentially leads to the release of pro-inflammatory mediators that could negatively influence fetal neurodevelopment. In this regard, several studies have evaluated the effect of MIA on the risk of ASD development. Some of these studies will be discussed in the following sub-sections of this review.

### 5.1 Maternal episodes of fever

One of the main observable effects of immune activation associated to infection is fever. Fever is an increase in body temperature resulting from the action of molecules called pyrogens in the hypothalamus. These molecules can be prostaglandins or cytokines produced by monocytes/macrophages in response to external stimuli such as infections, but also associated to environmental changes, and trauma. Also known as pyrexia, fever has an adaptive function of unique importance and is a potent activator of the immune system. Due to its role in the maintenance of physiological homeostasis through immune activation, fever can be considered as being part of the host defense mechanisms [69].

Immune outcomes related to body temperature elevation include lymphocyte activation and proliferation, accelerated neutrophil migration, and cytokine production - including interferon [70]. However, body temperature elevation (hyperthermia) due to fever or external heating during pregnancy has been associated with deleterious and teratogenic effects on the fetus. Those effects range from fetal loss to several malformations and impairments in development, especially considering brain development [71].

Being a common symptom during infections, and associated to a wide range of different pathogens, the presence of fever during pregnancy, even in the absence of intrauterine local infections [72], has been suggested as an important risk factor for ASD development in the offspring. In this sense, Zerbo et al. (2013) [16] investigated the possible association between maternal influenza virus infection and ASD incidence. Maternal infection was not associated with ASD, but the authors found an association between the occurrence of maternal fever during pregnancy and ASD. Interestingly, maternal use of antipyretic medication did not modify the results of the association between fever and impaired development [16]. These results corroborated previous findings [73] suggesting an association between ASD development and exposure to “high temperatures” during pregnancy.

Atladóttir et al. (2012) [74] reported an association of maternal fever with ASD risk after febrile episodes lasting seven days or more. This study also observed a two-fold increased risk of ASD in children from mothers that suffered from influenza virus infection. A prospecting study conducted in Norway evaluated the incidence of fever in pregnant women and ASD risk in 114,500 children born between 1999 and 2009. The development of ASD in children was associated with the occurrence of fever in the second trimester of pregnancy [75]. Finally, Anton et al. (2021) [76] reported results of a systematic review and meta-analysis exploring the association between prenatal exposure to fever and neurodevelopmental disorders outcomes in the offspring, providing additional evidence supporting such association. Taken together, the set of results mentioned above points to an association between febrile episodes during pregnancy and an increased risk of ASD. However, while the elevation of fetal body temperature associated with febrile episodes *per se* might be a risk component of fetal development disorders such as ASD, it always comes together with the release of inflammatory markers and immune mediators that could also play important roles in fetal development disorders, as will be addressed in the following sub-sections.

### 5.2 Cytokine unbalances during pregnancy

The investigation concerning the role of cytokines in the context of MIA is critical for the comprehension of ASD etiology. The term “cytokines” encompasses interleukins, chemokines, interferons (IFNs), tumor necrosis factors (TNFs), and growth factors. These molecules are small proteins ranging from 8 to 25 kDa, which play essential roles in several physiological processes, including cell growth, proliferation of neuronal tissues, as well as modulation of host immune responses to infection, injury, and inflammation [77].

During inflammatory processes, several pro-inflammatory cytokines (e.g. IL-1, IL-6, and TNF) are produced at the injury site by macrophages, lymphocytes, and other cells leading to local effects such as endothelial activation and immune cell activation and recruitment. Cytokines can also act systemically, inducing physiological changes in cells and organs distant from the tissue of origin. Examples of such actions are fever induction, by acting at the hypothalamus, and acute-phase protein synthesis induction in the liver. Excessive cytokine production during infection or injury could act at the maternal-fetal interface and influence fetal development. *Ex-vivo* placental perfusion experiments have shown that bidirectional transfer of cytokines across the placenta is possible, at least for some cytokines such as IL-6 [78]. Alternatively, mother-derived cytokines could act on cells at the maternal-fetal interface inducing secretion of cytokines and other factors that would reach the fetus brain and influence neurodevelopment.

In an experiment using a MIA rat model, the administration of IL-6 during pregnancy induced ASD-related symptoms in adult offspring, in contrast to the effect observed in offspring of *IL-6* knock-out rats injected with poly (I:C) – that mimics a viral infection. This study indicated that pro-inflammatory cytokine production during pregnancy, *per se*, has the potential to influence ASD etiology [79]. Additional findings of studies with animal models suggested that high levels of pro-inflammatory cytokines (e.g.: IL-2, IL-6, and IL-17a) during pregnancy were associated with a higher risk of ASD development in the offspring [80,81].

In humans, increased levels of circulating pro-inflammatory cytokines, granulocyte-macrophage colony-stimulating factor, IL-1α, IL-6, IFN-γ, and IL-1β in pregnant women had been associated with impaired intellectual abilities in the offspring diagnosed with ASD [82]. Of note, samples evaluated in this study were from women who participated in the state-mandated prenatal expanded alpha-fetoprotein screening program (XAFP) in Orange County, California – although no data about concomitant gestational disturbances is available [82]. Through amniotic fluid analysis of pregnant mothers of children with ASD and controls with typical development, Abdallah et al. (2013) [83] observed higher levels of TNF-α and TNF-β in mothers of affected children. Despite the findings pointing to a pivotal role of pro-inflammatory cytokines in mediating the effects of MIA on fetal neurodevelopment [84], there is no consensus regarding the mechanistic action of these molecules, or about their ability to cross the placental barrier [85,86]. Addressing these issues, a study evaluated amniotic fluid samples in mothers of Danish individuals diagnosed later in life with ASD and controls. Elevated levels of monocyte chemoattractant protein-1 (MCP-1/CCL2) were observed in mothers of children with ASD compared to controls [87].

Another study evaluated serum levels of 17 different cytokines in pregnant women whose children were classified in three distinct groups: ASD, other developmental disabilities, or presenting typical development. Significant higher concentrations of IFN-γ, IL-4, and IL-5 during pregnancy were identified in the group of mothers of children later diagnosed with ASD [88]. Elevated levels of IL-4 and IL-5, classified as anti-inflammatory cytokines, in the serum of mothers who gave birth to children later diagnosed with ASD further indicate that cytokine unbalances – either pro or anti-inflammatory – should be taken into account in the study of altered immune responses during pregnancy as risk factors for ASD manifestation in the offspring.

As outlined in section 1, cytokine unbalances, are not limited to the gestational period. Such alterations are also observed in ASD children and adults with the disease, as part of the neuroinflammatory status characteristic of autistic patients. These observations further indicate the relevance of the effects of the immune component in the broad spectrum of ASD clinical manifestations [[38], [39], [40]].

The management of immune dysfunctions has already been addressed and extensively discussed as a promising treatment strategy for ASD [89]. In this sense, treatment proposals include intravenous immunoglobulin (IVIG) infusion [90], corticosteroid therapy [91], and vitamin D supplementation [92]. The efficacy of IVIG infusion has recently been demonstrated in ASD children with immune impairments [93], and positive effects concerning improvements in language and behavior in young autistic children have also been achieved after corticosteroid therapy [94]. However, the potential benefit of using vitamin D supplementation in the treatment of ASD requires further investigation. These findings highlight the importance of the identification of specific ASD endophenotypes [89]. In this sense, integrative approaches, involving the characterization of inflammatory biomarkers, immune subtypes of cells, circulating cytokine profiles, and gastrointestinal status combined with genetic association studies, could be useful for personalized and more efficacious approaches. Mitigation of epilepsy and seizures could also be target with immune-related approaches since an immunological background has been postulated to these manifestations [95].

It is still unknown whether the unbalance in the level of circulating cytokines observed in autistic individuals after birth is a direct consequence of maternal immune changes to which these individuals may have been exposed during fetal development or if it is just a clinical manifestation of the disorder. Vargas et al. (2005) [45] evaluated autopsy-derived brain tissue samples from autistic patients, as well as cerebrospinal fluid of living patients. Different techniques determined the cytokine expression profile as well as the magnitude of neuroglial and inflammatory reactions in those samples. An intense neuroinflammatory process was identified in the cerebral cortex, white matter, and cerebellum of autistic patients. The study revealed neuroglia-derived macrophage chemoattractant protein (MCP)–1 and tumor growth factor as the most abundant cytokines in the tissues analyzed [45].

Li et al. (2009) [52] evaluated components of the immune response in brain tissue samples from individuals with ASD. Compared to matched controls with typical development, samples from autistic individuals showed increased levels of IL-8, IL-6, GM-CSF, IFN-γ, and TNF-α. Besides, the Th1/Th2 ratio in individuals with ASD was significantly polarized towards an inflammatory Th1 profile and, interestingly, the expected compensatory increase in IL-10 levels was not observed in those samples [52]. Such compensation via IL-10 production is expected because inflammation is usually composed of two phases and is characterized by a rapid production of pro-inflammatory factors, followed by a decrease in their release and subsequent delayed production of immunosuppressive mediators that limit their production or effect, thus avoiding host tissues harm. Of note, among the anti-inflammatory factors, IL-10 is considered as the quintessential immunosuppressive cytokine produced in CNS [96]. Furthermore, high levels of C-reactive protein in pregnancy have already been associated with ASD development in the offspring, representing an additional marker of exacerbated maternal inflammation [97].

A pivotal impact of the immune system on CNS development and functioning is supported by a robust body of evidences [98]. However, although specific interactions between these two systems, and the period and sequence of events in which they occur are still being elucidated, it is certain that cytokines and chemokines are key participants in such interactions. These molecules act directly on cells or affect the action and migration of different cells of both immune and nervous systems, such as microglia [99] and CD8^+^ T regulatory memory cells residing in the brain [100]. Thus, besides their role in inflammatory responses, cytokines are also “the common language between the nervous and the immune systems” [101]. For example, IL-1β and IL-2 receptors activate and modulate pathways such as those directed by mitogen-activated protein kinase (MAPK) and phosphoinositide 3-kinase (PI3K), both involved in CNS development and damage repair mechanisms [77].

### 5.3 Maternal hormone levels during pregnancy

Alterations in hormone levels have been suggested as contributing factors to ASD development when this condition is viewed through the perspective of an “extreme male brain”. In brief, this theory is based on demonstrations that autistic individuals show a masculinized shift in scores on two key sexually dimorphic psychological traits: empathy and systemizing behavior. These characteristics were suggested to be related to prenatal exposition to elevated testosterone levels in the womb [102].

In a study conducted in India, the parents of 942 children (471 diagnosed with ASD and 471 controls) answered a questionnaire, validated as a psychometric instrument, regarding maternal hormonal interventions during pregnancy. The study revealed that 58 out of 471 mothers from ASD cases, (12.3%) had undergone hormonal interventions during pregnancy, while this number dropped to 22 out of 471 among mothers from the control group (4.6%). The hormonal interventions by the Indian woman were injections of estrogens or progesterones as well as oral drugs such as human menopausal gonadotropin (hMG), Clomifene (an estrogen receptor modulator), and gonadotrophin-releasing hormone agonists (GnRH) [103].

A study addressed pre- and post-natal sex steroid hormone effects on autistic traits in children at 18–24 months of age. The levels of fetal testosterone and fetal estradiol were measured in the amniotic fluid of 35 pregnant women. Also, levels of post-natal testosterone saliva samples were collected from their children when they reached three to four months of age. The results showed a positive association between fetal testosterone levels with scores on the Quantitative Checklist for Autism in Toddlers (Q-CHAT), which was completed by the mothers and represents a measure for autistic traits [104]. In this same direction, high levels of androstenedione, the precursor of testosterone was observed amongst autistic women [105]. Extending the findings of elevated prenatal steroidogenic activity in ASD [106], a previous study has shown that prenatal estrogens contribute to autism likelihood, affecting both brain development and functioning and, ultimately, sexual differentiation [107]. However, these concepts are still under intense debate [108], and further investigation is needed.

## 6 Inflammation-related gestational disturbances as risk factors for ASD development

### 6.1 Gestational hypertension

The increase in blood pressure during pregnancy warrants closer monitoring of maternal and fetal health. Hypertensive diseases represent a global public health problem that affects up to 10% of pregnant women [109]. The definition of hypertension in pregnancy varies accordingly to international guidelines and includes different clinical phenotypes that are often assigned as transient [preeclampsia (PE), gestational hypertension] or chronic (pre-existing hypertension), depending on the onset of symptoms [110]. Noteworthy, irrespective of pre-existing or *de novo* hypertension, the association of hypertensive disorders with ASD is still a complicated feature since hypertension is a common symptom underlying many pathological alterations during pregnancy. Nevertheless, children exposed to hypertension during pregnancy are twice as likely to develop ASD [111].

In this context, PE is the most frequent hypertensive disorder of pregnancy (HDP), affecting 2–8% of pregnant women [112]. High blood pressure and inflammation are PE hallmarks, usually accompanied by several clinical manifestations affecting both maternal and fetal health [25]. Although much effort has been made to understand PE pathophysiology, delivery induction remains the only effective “treatment”. Besides the significant burden on public health, PE is responsible for up to 40% of preterm births [113]. Of note, a higher ASD prevalence has been reported in preterm as compared to in term infants [114,115].

The association between PE and ASD risk has been evaluated in several meta-analyses [24,116,117]. PE impacts on different ways both maternal and fetal health. For example, an increased risk for vascular related-disorders later in life and postpartum depression is observed among women previously diagnosed with PE [118]. Also, PE may compromise fetal neurodevelopment due to exposure to maternal systemic inflammation, to insulin resistance, nutrient deprivation, and chronic hypoxia [119]. Intrauterine hypoxia may affect the hypothalamic-pituitary-adrenal axis, increasing the risk for intrauterine growth restriction, cardiovascular disorders, and immunological imbalances in the fetus [120]. Altogether, exposure to hypertensive disorders of pregnancy may increase the risk for bronchopulmonary dysplasia, cerebral palsy, developmental delay, and ASD [119,121,122].

Identifying a causal link between PE and ASD is challenging since the association between ASD and PE is likely influenced by prematurity, birth weight, and other pregnancy-related factors [119]. Potential connections between PE and both inflammation and prematurity in neurodevelopment have been suggested and discussed [98,119]. In this context, exposure to maternal systemic inflammation may alter microglial activation (removal of cellular debris and synaptic pruning) and could lead to an increased ASD risk in the offspring [98]. Specific roles of inflammatory mediators during pregnancy in ASD susceptibility will be further reviewed in the following sections.

### 6.2 Gestational diabetes mellitus

Gestational diabetes mellitus (GDM) is a metabolic disorder characterized by hyperglycemia during the second or third trimester of pregnancy. GDM usually resolves following delivery [123]. Despite a substantial contribution of genetic factors [10], early pregnancy exposure to certain environmental factors was also associated with an increased ASD risk [124,125]. Intrauterine exposure to hyperglycemia has been consistently associated with ASD [9,36,[126], [127], [128]]. Also, the association of ASD with diabetes is highest for diabetes mellitus (DM) type 1, followed by DM type 2 and GDM when diagnosed by 26 weeks of gestation. However, when GDM is diagnosed after 26 weeks, the risk of having a child with ASD does not differ from the general population, thus implying that not only hyperglycemia but also the timing of exposure is an important factor associated with ASD risk [129].

Increased glycemia at 24–28 weeks of gestation increases the risk of adverse maternal, fetal and neonatal outcomes, even if values were kept within normal glycemia ranges for pregnancy [123,130]. Intrauterine hyperglycemia could impair fetal neuronal migration and connections or even lead to long-lasting epigenetic modifications in neuronal cells. These phenomena increase the complexity surrounding ASD development, adding to the presence of rare single nucleotide polymorphisms (SNPs) in idiopathic ASD and also to the low concordance, less than 50%, for ASD in monozygotic twins [9,131,132].

GDM does not only increases the risk of ASD, but also increases the risk of DM type 2, cardiovascular disorders in the offspring, and birth complications [133]. Importantly, it is widely known that obesity or elevated body mass index is a risk factor for DM and PE, both factors associated with ASD risk [123,134]. Moreover, chronic inflammation in obese individuals contributes to metabolic dysfunction [135]. Such physiological conditions during pregnancy *per se* or in association with gestational disorders could predispose to ASD development in the offspring [136].

Even during healthy pregnancies, there is an increase in the overall exosome level in the plasma of pregnant women [137]. Aiming to elucidate the effect of placenta-derived exosomes in GMD, a study compared the gestational-age profile of these nanovesicles in maternal plasma of GDM with pregnancies without complications. As expected, exosome concentration increased across gestation, but the study revealed that this increase was significantly higher in GDM cases [138].

### 6.3 Maternal overweight and obesity

No longer considered an inert tissue only devoted to storing energy, the adipose tissue has emerged as an active participant in the regulation of physiological and pathological processes, including immunity and inflammation. Adipocytes produce and release several molecules called adipokines (leptin, adiponectin, resistin, and visfatin), as well as pro- and anti-inflammatory cytokines [139]. Of note, obesity-associated inflammation in pregnancy has already been suggested to potentially influence the risk of ASD development [140].

Overweight and obesity are multifactorial conditions resulting from environmental, socioeconomic, biological, and genetic factors [141]. Besides, obesity should be considered a condition involving a strong inflammatory state. Excessive accumulation of lipids in the adipose tissue creates a pro-inflammatory environment in the body, inducing the production of inflammatory molecules such as IL-6, leptin, MCP-1, resistin, and TNF-α while reducing the production of adiponectin, leading to oxidative stress [142].

Considering the potential consequences of an uncontrolled inflammatory state during pregnancy, prenatal environmental factors such as maternal adiposity may influence the risk of ASD development. Maternal obesity and excessive gestational weight gain would result in overnutrition of the fetus, ultimately contributing to obesity and metabolic disturbances in the offspring [143]. Of note, evidence indicates that children with ASD were more likely to have mothers with higher weight gain during pregnancy than typically-developing children. Moreover, ASD risk could be increased in cases where mothers already had overweight before pregnancy [136]. However, further studies are needed to elucidate this aspect.

## 7 Maternal production of antibodies against the fetal brain

Another interesting aspect concerning the connection between the immune system and ASD-related symptoms is the identification of maternal antibodies against fetal brain proteins. Such antibodies, derived from women who had autistic children, were collected and injected in pregnant mice. As a result, the offspring of mice showed altered exploratory behavior and motor coordination, among other abnormalities [144]. In the same mice model, prenatal exposure to sera with antibodies from mothers of autistic children provoked anxiety disorders, startle reflexes, and lower sociability [145,146].

Studies using rhesus monkeys submitted to prenatal exposition to human immunoglobulin G (IgG) derived from mothers of ASD children presented stereotypies, hyperactivity [147], or impaired social behavior [148]. Although there is still little evidence, antibodies against fetal brain proteins are found in approximately 12% of women who had autistic children [149], and additional evidences demonstrated the specificity of these antibodies against 37 and 73 kDa brain proteins using IgG from murine models [150].

## 8 Maternal microbiota

The microbiota is suggested as a link between the immune system and prenatal environmental factors that contribute to ASD. In the gastrointestinal tract, resident bacteria form extremely complex ecosystems, and microbial metabolites play major roles in regulating the immune system and neural development. Thus, for the typical CNS development, it is believed that the correct maintenance of diversity and the prevalence of certain species of these microorganisms are necessary, both in the prenatal (maternal microbiota) and post-natal periods (newborn microbiota) [140,151,152].

The maternal gut microbiome composition contributes to obstetric outcomes with long-term health consequences for both the mother and the newborn [153]. It is speculated that the maternal diet during pregnancy impacts the levels, presence, or absence, of important immunomodulator molecules ultimately required for a successful pregnancy [151].

The expression “Brain-Gut Axis” has been used since the discovery of links between the brain and the gastrointestinal tract. The immune system is known to exchange information with the brain bi-directionally, through the autonomic nervous system, the enteric nervous system, and the hypothalamic-pituitary axis. In a healthy individual, all these pathways act in synchrony during CNS development. An imbalance in the number or diversity of microorganisms in a body compartment is called dysbiosis. Given the two-way nature of these complex connections, imbalances in dysbiosis-related inflammatory responses have potential impacts on features as diverse as CNS functioning, body weight, immunity, and behavior [153,154].

The gestation period is marked by profound changes in the maternal immune system [4]. These changes affect the pregnant woman's body in different ways, both locally as well as systemically. In this context, the maternal microbiota is extremely affected during pregnancy. The microbiota composition influences the entire gestation period and impact the health of both mother and fetus, even after childbirth [154].

Hsiao et al. (2013) [155] raised the critical role of the microbiota in addressing a mouse MIA model. Firstly, they demonstrated that gastrointestinal barrier defects and microbiota alterations in a MIA mouse model are linked to ASD features in mice offspring. Interestingly, oral treatment of MIA offspring with the human commensal *Bacteroides fragilis* was able to correct gut permeability, altering microbial composition, thus ameliorating disturbances in communicative, stereotypic, anxiety-like and sensorimotor behaviors. In addition, such treatment with *B. fragilis* was able to reestablish IL-6 levels along with the attenuation of symptoms [155].

Concerning ASD-diagnosed individuals [156], analyzed both bacteria and fungi gut microbiota. The study showed an increase in the Firmicutes/Bacteroidetes ratio in autistic subjects due to a reduction of the Bacteroidetes relative abundance. Considering fungi, the relative abundance of the genus *Candida* was more than double in autistic than neurotypical subjects [156]. Based on the genome-wide association study of gut microbiota (GWASGM), a search for significant genetic associations between host genes and gut microbiota composition was made in the context of different psychiatric disorders. Regarding ASD, association signals were observed for the genus *Bacteroides* and *Desulfovibrio* [157]. In agreement, *Desulfovibrio* species were previously found in significantly higher numbers in the stools of severely autistic children as compared to controls [158,159].

## 9 Labor type and labor-associated factors

Childbirth is a process marked by extreme physiological stimuli for both mother and newborn. Along with the physiological events that permeate labor, the expulsion of the baby out of the mother's body is a major physical stress. Considering physiological characteristics, birth is stressful and could be even a traumatic event for both mother and fetus [160].

Some studies have shown that childbirth is a process that may influence the occurrence of ASD. Considering that labor is a physiological process involving inflammatory responses [161], it is interesting to note that a longer period of normal labor has already been observed in mothers of children who developed ASD [73]. In addition, forceps-aided delivery has been associated with ASD [18]. A case-control study addressing all singleton births in Finland from 1990 to 2005 investigated the relationship between obstetric risk factors and childhood autism, Asperger syndrome, and other pervasive developmental disorders. Of note, birth type (vacuum extractor or forceps) was associated with childhood autism. However, this statistical association was lost after adjustment for potential confounders [162].

Highlighting aspects sometimes neglected, a study evaluated fetal exposure to the so-called “labor and delivery drugs” along with other labor and delivery risk factors [163]. The results revealed that children born from mothers who used a drug or combination of drugs that induce labor have a higher risk of ASD development [163]. A follow-up study addressing all live births in Sweden between 1992 and 2005 investigated the association between labor induction and ASD. In this sample, 11% of all live births were preceded by labor induction and 1.6% of individuals were diagnosed with ASD. However, the association of labor induction and ASD was not significant when a comparison was performed within siblings with discordant labor induction, thus suggesting that concerns about ASD risk should not influence the clinical decision about labor induction [164].

Several studies have indicated an association between cesarean delivery and ASD development in children from different countries [162,165,166]. Besides [167], revealed an association between ASD risk and cesarean delivery with general anesthesia. Interestingly, the analyses from this study revealed that the observed association between cesarean section with general anesthesia and ASD remained statistically significant only among cases of children severely affected with ASD. In the same direction [168], reported that neonates delivered by cesarean section with general anesthesia were more likely to be diagnosed with ASD as compared to those exposed to vaginal birth or cesarean section with regional anesthesia.

Attempts to explain the association between cesarean delivery and ASD incidence include different hypotheses. One explanation involves a deregulation of optimal oxytocin levels since perinatal imbalances in oxytocin levels may result in adverse effects on childhood and adulthood [169]. Oxytocin participates in the regulation of uterine contractions during labor and also influences the efficiency of birth. Regarding childbirth, oxytocin is released in pulses, gradually increasing its concentration in maternal circulation, with a maximum peak recorded in the first hour after birth. A direct consequence of planned cesarean section surgery is the absence of exposure of the fetus to these hormone levels. In addition, oxytocin participates in biochemical processes related to mood and social behavior. Supporting this view, decreased levels of oxytocin are detected in the plasma of individuals with ASD compared to typically developing controls [170].

Moreover, oxytocin is a hormone with important participation in the regulation of social skills and interactive activities. Considering the birth-childbirth-development of ASD, Ben-Ari (2015) [171] suggested that childbirth is a critical moment of stress, responding by the attenuation or aggravation of potential deleterious effects that occurred during pregnancy (due to genetic or environmental factors). In addition to issues related to oxytocin and general anesthesia, the type of delivery may impact the individual's microbiota throughout childhood and adulthood; and this factor may influence the risk of ASD development or the disease pathogenesis.

## 10 Extracellular vesicles and ASD: an emerging topic

The expression “extracellular vesicle” (EV) encompasses several lipid-enveloped structures released by cells of eukaryotic and prokaryotic organisms through shedding mechanisms. EVs receive different names according to size, shedding mechanism, shape, and content, including “microparticles”, “microvesicles”, “nanovesicles”, “nanoparticles”, “ectosomes”, “exosomes”, “exovesicles”, and “exosome-like vesicles” [172]. The diversity regarding the origin and function of these several vesicle types requires careful characterization of EVs during experiments. Evaluation of size, shape and biochemical composition is strongly recommended for identifying EV subtypes and functions [173]. Regarding EVs classification as, we should keep in mind the recommendation of MISEV2018 (Minimal information for studies of extracellular vesicles 2018) which states that exosomes and microvesicles are “historically burdened by both manifold, contradictory definitions and inaccurate expectations of unique biogenesis” [173]. While some articles discussed here have not conducted sufficient or adequate experimentation to disclose the origin and exact classification of EV type, we have chosen to use the terminologies presented in the original publications.

**Fig. 3:**
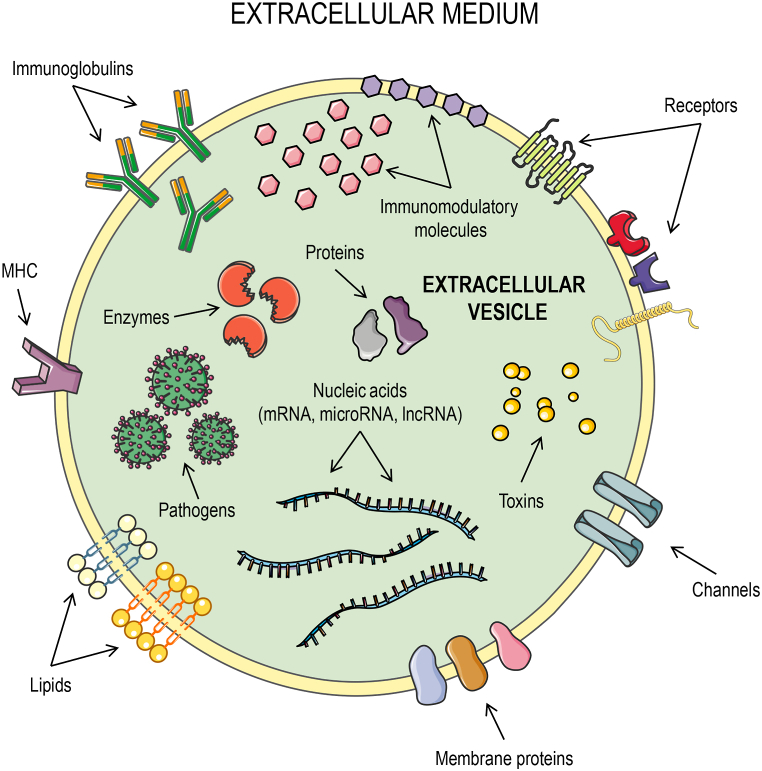
Representation of a hypothetical extracelular vesicle (EV) and its different cargoes. Various types of molecules can be found on the membrane or within EVs released by mammalian cells, including membrane receptors, lipids, intracellular and membrane proteins, membrane channels, toxins, nucleic acids (mRNA, microRNA, lncRNA), enzymes, pathogens, immunoglobulins, MHC, and immunomodulatory molecules. This figure was created using *Servier Medical Art* illustrations (available at https://smart.servier.com, under a Creative Commons Attribution 3.0 Unported License).

In multicellular organisms, EVs have been isolated from basically all biological fluids, including blood, urine, synovial fluid, saliva, breast milk, amniotic fluid, broncho-alveolar lavage fluid, ascites, cerebrospinal fluid, bile, vaginal fluid, and semen [172,174]. Despite the common features used for EV identification and characterization, it is important to emphasize that EV-associated cargoes differ depending on the organism, cell or tissue of origin. Fig. 3 illustrates a hypothetical EV of mammal origin, showing the various types of molecules that can be found on the membrane or within mammalian EVs, including membrane receptors [173], lipids [175], membrane channels [176], pathogen-derived toxins [177], nucleic acids [178], pathogens [179], immunoglobulins [180], and immunomodulatory molecules 181].

Different types of EVs can cross physical and physiological barriers and perform essential roles in cell-to-cell communication. In this sense, EVs are critical modulators of the immune response in both healthy and pathological backgrounds [4,182,183]. The use of EVs as carriers of immunoregulatory molecules is proposed as promising therapeutic tool targeted to different conditions [[184], [185], [186]].

Exosomes are a specific group of EVs showing 40–100 nm in size and generated by endocytic pathways. These nanovesicles are highly implicated in pregnancy, being intensely secreted by placental cells and playing important roles in the immunomodulation at the maternal-fetal interface. This communication between mother and fetus via EVs runs from the syncytiotrophoblast towards maternal [181,187] and modifies susceptibility to various [4]. The concentration of exosomes in the circulation of pregnant women increases over the gestational period, and correlates with uterine blood flow and placental weight at delivery [137]. Regarding gestational disorders such as PE, the number of circulating exosomes is quite higher, and has been suggested as a potential tool for pregnancy monitoring [188].

Upon stimulation, neurons release exosome-like EVs. The release of neurotransmitters by these nanovesicles likely represents a mechanism of protein sorting and quality control disposing of such receptors or regulating excitability in a lysosome-independent manner. Experiments involving cell depolarization of differentiated neuroblast cultures have shown that these cells contain microRNAs that are released as exosome cargoes, which ultimately could be involved in silencing regulation during cellular events, such as synaptic plasticity. Besides neurons, different cells of the nervous system act in cooperation with exosomes and EVs in general, such as oligodendrocytes, Schwan cells, astrocytes, and microglia [189]. Microvesicles intensely participate in neuroinflammation processes, being suggested as biomarkers in different CNS diseases [190,191]. For instance, patients with multiple sclerosis have shown increased numbers of microvesicles in blood and cerebrospinal fluid. Moreover, microglia and astrocytes can spread inflammatory signals by the release of EVs associated with inflammatory molecules, such as IL-1β, IFN-γ, TNF, caspase 1, and the P2X7 receptor, among other factors [189]. In addition, since EVs can reach the brain by crossing the blood-brain barrier, EVs from outside the CNS could exert their effects directly on CNS cells [192]. This characteristic has implications for neurophysiologic disease modeling and treatment strategies.

It has been established that EVs control immune responses. Also, EV cargoes secreted in the intercellular space by different cell types can act as triggers of microglia activation [193]. Along with strong indications that exosomes deliver biological information to neurons [189,194,195], there is growing evidence pointing to a role of EVs in psychiatric conditions, including ASD [5,[196], [197], [198]]. Tsilioni and Theoharides (2018) [5] reported a significantly increased concentration of EVs in serum of children with ASD as compared to healthy normotypic controls. Such EVs presented as cargoes mtDNA, which are important triggers of inflammatory response both *in vivo* and *in vitro* [199]. Furthermore, EVs containing mtDNAs have shown the ability to stimulate human-cultured microglia to secrete the pro-inflammatory cytokine IL-1β, potentially acting in the brain of children with ASD [5]. In this direction, significantly higher mtDNA levels in serum of autistic individuals compared to normotypic controls were already reported [200].

The hypothesis that EVs could drive neuroinflammation deserves additional explanations, especially concerning the signals for the synthesis of inflammatory mediators. It was proposed that EV-mediated IL-1β production via inflammasome would involve mtDNA as a first signal and the neuropeptide neurotensin as a second signal. Such proposal is based on findings pointing to the neurotensin capacity to stimulate microglia to secrete IL-1β [201] along with the observation of elevated levels of both IL-1β in the brain [202] and neurotensin in the serum [203] of children with ASD.

Intranasal exosome-based therapies have been suggested for the treatment of neuronal disorders [204,205]. Aiming to track the migration and homing patterns of intranasally administrated exosomes derived from bone marrow mesenchymal stem cells in a set of brain pathologies, including stroke, autism, Parkinson's disease, and Alzheimer's disease [206], presented a mechanism for longitudinal and quantitative *in vivo* neuroimaging of exosomes. This approach was based on the superior visualization abilities of classical X-ray computed tomography associated with gold nanoparticles as labeling agents [206].

Mesenchymal stromal cells (MSCs)-derived exosomes were proposed as a promising strategy to prevent perinatal brain injury in human preterm newborns [205]. MSCs can ultimately avoid the activation of brain resident immune cells, protecting brain tissues against inflammation-related damages. It was demonstrated that intranasal application of MSC-derived exosomes before ischemia significantly prevented perinatal brain injury in a rat model [ 205]. The support for these therapeutic strategies also came from a model of inflammation-induced preterm brain injury [207]. Using an *in vitro* model of oxygen-glucose deprivation/reoxygenation mimicking hypoxic-ischemic injury in the mouse neuroblastoma cell line neuro2a [208], addressed the effects of human Wharton's jelly MSC (hWJ-MSC)-derived EVs on neuroprotection and neuroregeneration. The overall results indicated that the EVs derived from hWJ-MSC can prevent and resolve apoptosis in neuronal cells in the immature neonatal brain that was induced by hypoxic-ischemic insults. More specifically, the antiapoptotic effect of hWJ-MSC seems to be mediated by the transfer of EV-derived let-7-5p miR [208]. Besides, results from treatment with MSC-derived exosomes via intranasal administration showed significant behavioral improvement of both genetic and idiopathic autism features in mice [209].

As previously discussed, existence of a traffic of EVs from the placenta to the maternal circulation is already a consensus. In the opposite direction, the traffic of biological components from mother to fetus has not been the focus of many studies, but some information can be obtained from papers published decades ago. Although we should be concerned by ethical issues (many ethical standards accepted in the past are no longer accepted), such as the use of radioactive material in research, some of these early experiments demonstrated the passage of cells and platelets from mother to fetus during pregnancy, as summarized in Table 2 (in a historical perspective) [[210], [211], [212], [213], [214], [215], [216]]. Since there is a passage of different cell types from the maternal bloodstream towards the fetus, mother-derived EVs could also reach organs and systems of the fetus, including the CNS, impacting fetal development.

**Table 2:** Experiments showing the transference of factors from maternal blood towards the fetus, based on historical literature.

Method	Main findings	Reference
Red cells tagged with radioactive iron (Fe^59^) were infused in 7 pregnant women by autotransfusions.	The infants of 4 of these women presented radioactive erythrocytes on their bloodstream.	[210]^a^
Transfusion of sickle cell blood was performed in 25 pregnant women.	Transmission of transfused sickle-trait cells from the mother to the fetus.	[211]
Erythrocytes from 18 pregnant women were labeled with Cr^51^ and re-injected into these women before delivery.	Of 18 births, radioactive activity was detected in the blood of 13 umbilical cords.	[210]^a^
Autotransfusions of red blood cells tagged with Cr^51^ were done in 33 pregnant women with signals of preterm delivery.	Transmission of erythrocytes from mother to fetus was observed in 26% of cases.	[210]
Elliptocytic blood was infused in 2 pregnant women.	It was observed elliptocytes in the blood of one of the infants along with multiple sites of bleeding and infarction on the placenta.	[212]
Red cells tagged with radioactive iron (Fe^59^) were infused in 29 pregnant women. In addition, 2 other pregnant women received blood from a donor with the sickle cell trait.	From the experiment with Fe^59^, the infants of 25 women presented radioactive erythrocytes on their bloodstream. Also, sickle cells were observed in the infants of woman who received sickle cells.	[213]
Blood cells from 9 pregnant women were collected, exposed to Atabrine dihydrochloride and auto-transfused prior to delivery.	Fluorescent forms were detected in 6 umbilical cords out of the 9 infants born. Besides, fluorescence was detected in granulocytes and platelets in 4 cases and 3 cases present fluorescent lymphocytes.	[214]
Erythrocytes tagged with radioactive phosphorus (P^32^) were injected into 6 pregnant women prior to delivery.	A significant degree of radioactivity was observed in one of the infants.	[215]
Placentas from 44 pregnant women with sickle cell were analyzed where sickled erythrocytes served as a marker of maternal blood transferring to the fetus.	Concurrent incidence of sickle cells in maternal and fetal blood was observed in 100% of the cases. The passage of erythrocytes was four times more frequent in umbilical or chorionic veins than in arteries.	[216]

We intentionally cite some old references because we consider their scientific contribution still valid. Of note, these studies were carried out at a time with ethical and safety standards that are unthinkable today, as outlined in the text.^a^ cited by Zarou et al. (1964).

**Fig. 4:**
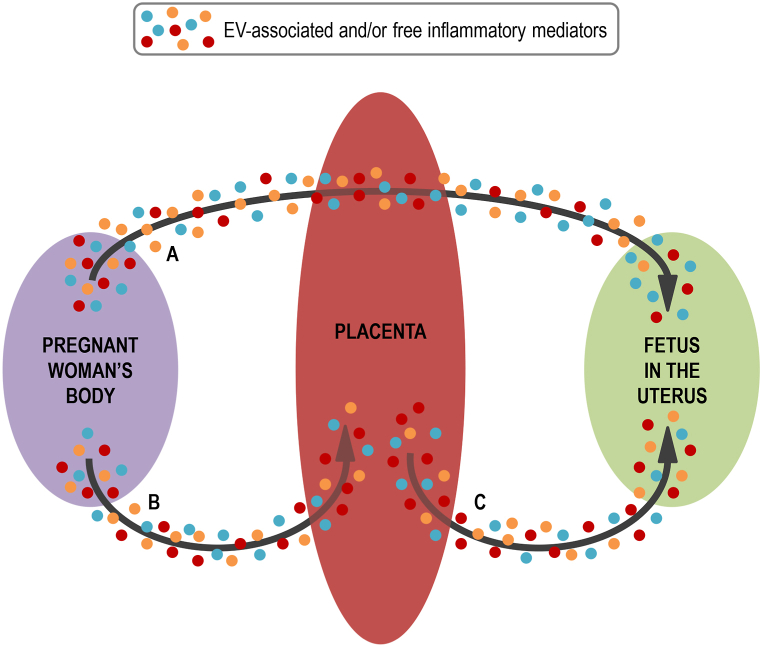
Potential routes of maternal-derived immunomodulatory/inflammatory molecules (Inf-molecules) towards the developing fetus. Inf-molecules may be transported free or coupled to extracellular vesicles (EVs). Inf-molecules/EVs can be directly transported from the maternal circulation to the fetus through the placenta (pathway A). Alternatively, Inf-molecules/EVs can reach the placenta through the maternal circulation (pathway B), stimulating the placenta to release other Inf-molecules/EVs that will exert their effects on the CNS of the developing fetus (pathway C).

Considering the scenario mentioned above and the potential impacts of exosomes and other EVs on autistic individuals, several questions emerge: (a) What is the origin of these exosome-like EVs? (b) What is the composition of these exosome-like EVs? (c) Do these exosome-like EVs interact with CNS cells? (d) Is it possible to isolate these increased exosomes-like EVs in autistic patients after infections? Not all these questions can be completely answered with scientific data presently available and, therefore, more research about these aspects is needed. Potential answers to some of these questions and hypotheses are shown in Fig. 4.

## 11 Combining the topics

Exosomes, as well as other EVs, participate in various immune processes, in both pro- and anti-inflammatory conditions associated with disease or healthy physiological processes, including pregnancy. Natural elevation in exosome concentration could be seen in the peripheral circulation of healthy pregnant women [137] along with an intense release of different EV types by the placenta at the maternal-fetal interface [187]. Both peripheral elevation of exosome concentrations and intense EV release by the placenta can lead to exosome-mediated immunosuppression through the control of apoptosis of activated lymphocytes with a potential of harming the fetal/placental development [181,217]. On the other hand, exacerbated levels of exosomes with pro-inflammatory characteristics are found in the plasma of women with PE [188]. Considering that PE is a risk factor for ASD [24,116,117], exosomes along with (or carrying) pro-inflammatory mediators could be a neglected mechanism in ASD etiology, mainly during gestation. In addition, the role of EVs in the communication of different cell types of the nervous system [189] and differences in concentrations of serum exosomes between individuals with ASD and normotypic controls [5] further suggest exosomes and other EVs as potential candidate components of ASD etiology.

Besides the genetic component of the disorders included in ASD, factors of the immune system (which are under the effect of the characteristic milieu associated with pregnancy) could be considered as triggers for ASD development. Besides the association of ASD with PE and other pregnancy related-variables, data obtained from different approaches lead to controversial results concerning which specific factors contribute to ASD development. Therefore, further studies with well-designed cohorts and robust methodological approaches are required to explain these aspects in a detailed way.

Epidemiological data of ASD is likely incomplete due to a lack of studies from developing countries where maternal-fetal mortality and morbidity are highly associated with vascular disorders. The body of evidence reviewed here strongly suggests that a proper development of the fetal CNS is directly dependent on the proper functioning of both the fetal immune and nervous systems, along with the correct homeostasis of other physiological systems in the maternal and fetal counterparts.

The dynamic interactions between fetal and maternal immune systems encompass maternal immune responses to different biological challenges during pregnancy [4,187]. EVs actively participate in the immune adjustments necessary for proper fetal development; and the abundance and content of these vesicles are directly affected by physiological changes during pregnancy, infections and disorders (e.g., PE). Furthermore, immunological alterations in the maternal body, including the responses generated by cytokines and chemokines produced in face of different challenges, enhance inflammatory responses at the maternal-fetal interface. Importantly, these responses can be either pro-inflammatory or anti-inflammatory, depending on the context in which such a response is induced.

In addition to the potential impacts of inflammation and MIA on fetal CNS development and ASD risk, inflammation is a common feature of individuals with ASD [89]. The presence of brain-reactive antibodies [42], abnormal T cell counts and function [218,219], altered cytokine levels in the brain, cerebrospinal fluid and bloodstream [[45], [46], [47]], elevated levels of circulating monocytes [48], and dysregulation in the activity of Natural Killer cells [49,50], are among the reported immune alterations observed in ASD individuals.

**Fig. 5:**
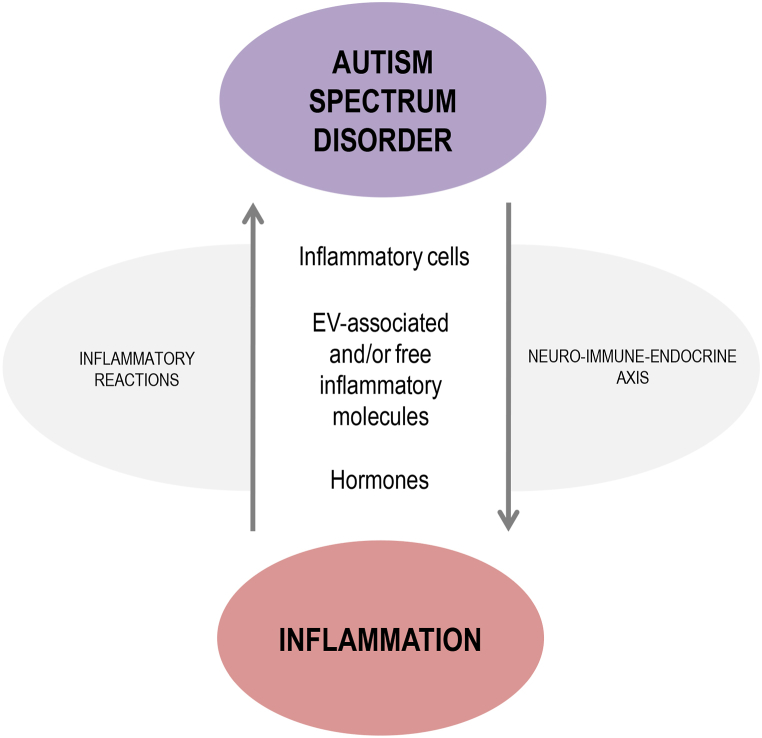
Interplay between autism spectrum disorder (ASD) and inflammation. In addition to the potential influence of inflammation/maternal immune activation on the development of ASD during development of fetal CNS, inflammation is a common feature of individuals with ASD. Inflammation may contribute to the maintenance of the phenotype and symptoms of ASD. Also, inflammation may be a consequence of dysregulations in the neuro-immune-endocrine axis associated with ASD. The relationship between ASD and inflammation can therefore form a feedback.

Considering these observations, inflammation (and perhaps a chronic inflammatory status) may contribute to the maintenance of the phenotype and symptoms of ASD. Also, inflammation may be a consequence of dysregulations in the neuro-immune-endocrine axis associated with ASD. The relationship between ASD and inflammation can, therefore, originates a feedback loop. Fig. 5 summarizes the interplay between ASD and inflammation. EV-associated inflammatory components, free inflammatory molecules, hormones, and inflammatory cells mediate the interactions between inflammation and ASD.

Various molecules participate in the regulation of the maternal immune system at the maternal-fetal interface, whose presence, absence, and abundance are highly influenced by maternal genetic factors [26,184,[220], [[221]], [222]] as well as by the genetic background of the fetus [223,224]. However, how gene variants (e.g., SNPs) of immune system-related genes influence the etiology of ASD has yet to be demonstrated.

Another interesting fact that deserves consideration is the increasing number of women using birth-inducing drugs in the last 30 years. Being a physiological process that also involves an inflammatory response, labor may be an additional trigger for immune responses, contributing to the risk of ASD development. Regarding cesarean sections, which involve programmed delivery, the absence of fetal exposure to the physiological stimuli underlying labor (especially oxytocin) is also of concern. Alternatively (or additionally), the possible link could be beyond cesarean delivery *per se*, resulting from differences in the microbiome established in children's guts following cesarean or vaginal delivery.

## 12 Conclusion

Classical evidence and recent discoveries reviewed in this article led us to suggest EVs as neglected components connecting inflammation and ASD development. The role of EVs as immunomodulatory factors during pregnancy [4,187], along with the classification of PE and GDM as risk factors for ASD development [9,111], and the increased levels of EVs observed in peripheral serum of pregnant women with PE and GDM [138,188] are some key evidences potentially linking EVs and ASD symptoms. Interestingly, EVs participate in microglia activation [189] and EV-associated proteins are indeed found in increased levels in the serum of children with ASD [5,196,197]. Thus, once EVs levels can be altered in ASD individuals, the potential of EVs reaching the brain and acting as triggers of inflammation in the context of ASD is an interesting hypothesis.

Taken together the evidence discussed in this review, we suggest EVs as important mechanistic drivers of the molecular triggers and enhancers of inflammation, ultimately influencing the risk of ASD development. Besides the ability of EVs to protect the inflammatory molecules they carry from degradation, EVs can also facilitate gestational intercurrences and should be considered as factors accounting for ASD development. Finally, we stress that the connections and hypotheses outlined in this review need complete investigation since a limitation in our proposals is the lack of clinical studies regarding EVs in ASD individuals, an issue also raised elsewhere [198].

## Funding

VLK received a doctoral scholarship from 10.13039/501100002322*Coordenação de Aperfeiçoamento de Pessoal de Nível Superior* (CAPES, Brazil). VLK receives a postdoctoral fellowship from 10.13039/501100001807Fundação de Amparo à Pesquisa do Estado de São Paulo (FAPESP); RTM, JBS and JHE receive postdoctoral fellowships from 10.13039/501100002322CAPES (Brazil, finance code 001). JABC receives research fellowships from *Conselho Nacional de Desenvolvimento Científico e Tecnológico* (*Bolsa de Produtividade em Pesquisa - Nível 1A*, 10.13039/501100003593CNPq, Brazil), Fundação de Amparo à Pesquisa do Estado do Rio Grande do Sul (FAPERGS, Brazil), and 10.13039/501100002322CAPES (AUXPE 686/2020, Brazil).

## Author contribution statement

All authors listed have significantly contributed to the development and the writing of this article.

## Data availability statement

No data was used for the research described in the article.

## Declaration of interest’s statement

The authors declare no competing interests.

## Declaration of competing interest

The authors declare that they have no known competing financial interests or personal relationships that could have appeared to influence the work reported in this paper
